# *Caenorhabditis elegans* Show Preference for Stimulants and Potential as a Model Organism for Medications Screening

**DOI:** 10.3389/fphys.2018.01200

**Published:** 2018-08-30

**Authors:** Eric A. Engleman, Kevin B. Steagall, Kristin E. Bredhold, Michaela Breach, Hannah L. Kline, Richard L. Bell, Simon N. Katner, Bethany S. Neal-Beliveau

**Affiliations:** ^1^Department of Psychiatry, Indiana University School of Medicine, Indianapolis, IN, United States; ^2^Department of Psychology, School of Science, Indiana University–Purdue University Indianapolis, Indianapolis, IN, United States

**Keywords:** addiction research, cocaine, nicotine, invertebrate models, self-administration models, high-throughput screening assays

## Abstract

The nematode *Caenorhabditis elegans* (*C. elegans*) is a popular invertebrate model organism to study neurobiological disease states. This is due in part to the intricate mapping of all neurons and synapses of the entire animal, the wide availability of mutant strains, and the genetic and molecular tools that can be used to manipulate the genome and gene expression. We have shown that, *C. elegans* develops a conditioned preference for cues that had previously been paired with either cocaine or methamphetamine exposure that is dependent on dopamine neurotransmission, similar to findings using place conditioning with rats and mice. In the current study, we show *C. elegans* also display a preference for, and self-exposure to, cocaine and nicotine. This substance of abuse (SOA) preference response can be selectively blocked by pretreatment with naltrexone and is consistent with the recent discovery of an opioid receptor system in *C. elegans*. In addition, pre-exposure to the smoking cessation treatment varenicline also inhibits self-exposure to nicotine. Exposure to concentrations of treatments that inhibit SOA preference/self-exposure did not induce any significant inhibition of locomotor activity or affect food or benzaldehyde chemotaxis. These data provide predictive validity for the development of high-throughput *C. elegans* behavioral medication screens. These screens could enable fast and accurate generation of data to identify compounds that may be effective in treating human addiction. The successful development and validation of such models would introduce powerful and novel tools in the search for new pharmacological treatments for substance use disorders, and provide a platform to study the mechanisms that underlie addictions.

## Introduction

The impact of addiction on our society is profound and by all accounts is increasing. It has been estimated that in the United States alone, addiction costs approach 200 billion dollars (U.S. Department of Justice, 2010). Clearly, there is an acute need for a better understanding of the neurobiological basis of addiction, as well as better and more effective treatments to confront this growing epidemic. Animal models have provided much of our current understanding about the neuroscience of addiction (Edwards and Koob, 2012). In particular, behavioral measures used to model and study human addiction in animals (cf., Bell and Rahman, 2016) in conjunction with functional neurobiological studies have provided us with an understanding of basic reward circuitry (Koob and Volkow, 2010). Development of pharmacotherapeutic medications is a promising avenue to reduce the impact of substance use disorders; however, few such treatments are currently available. Thus, additional efforts are needed to identify molecular targets and novel compounds for medications development. Work from our group and others shows that the effects of substances of abuse (SOAs) on neurobiology and behavior is phylogenetically ancient, suggesting that invertebrates possess some of the mechanisms that underlie addiction. Techniques historically used to study behavioral aspects of SOAs in vertebrates such as conditioned place preference (CPP) and SOA self-administration (Tzschentke, 2007) have also been developed for invertebrates. Elegant studies have shown SOA reward, withdrawal, and seeking to opiates, cocaine, and amphetamines in crayfish (Huber et al., 2011). In *Drosophila melanogaster*, ethanol (EtOH) conditioning and self-administration paradigms have demonstrated that flies develop conditioned preference responses to cues previously paired with EtOH (Kaun et al., 2011). These data show that even simple invertebrate animals can model what are widely considered to be highly complex behaviors. However, this should not be surprising as behavioral models using invertebrates have played a key role in discovering the underlying molecular mechanisms that provide the basis for learning and memory (Kandel, 2001).

The nematode *Caenorhabditis elegans* has some major advantages as a model organism to study neurobiology and disease states (Hulme and Whitesides, 2011). *C. elegans* have conserved neurobiological systems with established mapping of all neurons and synapses in the entire animal. We have shown that, *C. elegans* develops a conditioned preference for cues that had previously been paired with either cocaine or methamphetamine exposure that is similar to findings using place conditioning with rats and mice (Musselman et al., 2012; Katner et al., 2016). Moreover, conditioning required functional dopamine neurotransmission (Musselman et al., 2012). Additionally, with SOA pre-exposure, *C. elegans* demonstrate tolerance (Grotewiel and Bettinger, 2015) and sensitization (Lee et al., 2009) which are hallmarks of addiction in humans. Together, these data indicate that *C. elegans*, show behavioral responses to SOAs that are consistent with those of higher level organisms. These data also indicate that invertebrates, specifically *C. elegans* in this case, show behavioral responses to addictive SOAs that are consistent with those seen in more complex animals. Recent research has established that *C. elegans* display depressed locomotion and functional tolerance after contact with EtOH which is mediated, in part, through the BK potassium channel which may mediate behavioral sensitivity to EtOH in many species including humans (Bettinger and Davies, 2014; Davis et al., 2014). Importantly, the internal tissue concentration leading to the effects of EtOH on locomotor activity in *C. elegans* is strikingly similar to blood alcohol levels that produce intoxication in humans (Alaimo et al., 2012). These data suggest that *C. elegans* show a concentration-dependent attraction to EtOH that results in EtOH self-exposure and significant tissue concentrations of EtOH. We have discovered that this EtOH preference response can be selectively blocked by pretreatment with the pan-opioid receptor antagonist, a treatment for alcohol and opiate use disorders, naltrexone, which is consistent with the recent discovery of an opioid receptor system in *C. elegans* that mediates responses to both appetitive stimuli (Cheong et al., 2015) as well as nociception (Mills et al., 2016). In the current work, we have expanded the use of such a treatment approach on cocaine and nicotine preference and have examined the effects of the nicotinic cholinergic partial agonist, and smoking cessation treatment, varenicline, on nicotine preference (Gomez-Coronado et al., 2018). The results suggest an opportunity to establish and validate a high-throughput *C. elegans* behavioral medications screening model for stimulant addiction. The successful development and implementation of such models would provide the field with powerful and novel tools in the search for new pharmacological treatments for addictions, and provide a platform to study the underlying mechanisms of these agents.

**Objective**: To determine if *C. elegans* may be used to model the behavioral aspects of stimulant self-administration and to screen for putative addiction treatments.

## Materials and Methods

### Drugs

Cocaine hydrochloride was received from the NIDA Drug Supply Program, nicotine bitartrate was purchased from Sigma Aldrich (St. Louis, MO, United States), and varenicline tartrate was purchased from Biotang (Lexington, MA, United States). Vehicle (0.97 mM HCl; salt equivalent of naltrexone HCl) and naltrexone HCl (N-3136; FW 377.9; Sigma-Aldrich) were used to pretreat animals prior to SOA preference testing. Benzaldehyde (#418099; 99.5%; Sigma-Aldrich; FW 106.12) was used to test for non-selective effects of naltrexone HCl. 2-nonanone (99%; CAS 821-55-6; FW 142.24; Arcos Organics) was used to show that animals could move away from the SOA target zone.

### Culture and Maintenance of Strains

The N2 Bristol wild-type (WT) strain was used in all assays. All animals were maintained at 22°C, and all general culturing techniques have been described previously by Nass and Hamza (2007). Worms were grown with *E. coli* strain NA22 as a food source on maintenance plates, produced by filling 60-mm petri dishes with 10-ml regular NGM agar [25 g bacto agar, 20 g bacto peptone, 3 g NaCl, 1 L H20, 1 ml cholesterol (5 mg/ml 95% ethanol), 1 ml 1 M CaCl2, 1 ml 1 M MgSO4, and 25 ml of potassium phosphate buffer]. The potassium phosphate buffer contained 5 g of K2HPO4 dibasic/anhydrous, 30 g of KH2PO4 monobasic, and 500 ml of H20, pH adjusted to 6.0 (Bianchi and Driscoll, 2006).

Adult worms were used for all experiments to control for any effects of different sensitivities and responses to SOAs at varying developmental stages. Worms were age synchronized by lysing gravid adults with bleach and sodium hydroxide, allowing eggs to be released into solution and hatched in M9 buffer (Bianchi and Driscoll, 2006). After 18 h, hatched L1 larvae were washed three times with water, plated, and maintained on NGM plates with NA22 *E. coli* bacterial lawns until reaching adulthood. Testing began approximately 72 h post-plating the L1 larvae, when worms were adults.

6-well Costar^TM^ cell culture plates were used to determine SOA preference (Fisher cat. no. 07-200-80). Clear templates were taped to the bottom each 6-well plate to create two 1.2 cm diameter circular target zones within the 3.5 cm diameter of each well. Test plates were produced by filling each well of the plates with 3.8 ml of NaCl free agar (17 g bacto agar, 2.5 g bacto peptone, 1 L H20, 1 ml 1 M CaCl2, 1 ml 1 M MgSO4, and 25 ml of potassium phosphate buffer). Cholesterol was not included in the salt-free agar in order to obtain clearer images of worms during testing. Although the lack of salts and cholesterol in the agar may have long-term effects on worms, our previous work indicating intact cue-conditioned learning (Musselman et al., 2012; Katner et al., 2016) and the differential responses with the SOAs vs. controls (food or benzaldehyde) show that the agar preparation as used in this paradigm does not prevent normal chemotaxic responses.

### Treatment Agent Pretreatment Prior to SOA Preference Testing

Worms were washed off maintenance plates with 15 ml of water and transferred to 15 ml centrifuge tubes. Adults were allowed to settle on the bottom of each tube for 5 min and then the supernatant was removed. This was repeated two more times with 10 ml of water to remove the majority of bacteria from the worms. After the final removal of the supernatant, approximately 0.3–0.5 ml of worms were transferred to a 5 ml Eppendorf tube and 3 ml of vehicle (0.97 mM HCl) or treatment agent naltrexone HCl (10 mM; dose selected from Cheong et al. (2015), or varenicline (1.0 or 9.0 mM) was added to each tube. The tubes were placed on a nutator for 30 min prior to SOA preference testing. Following vehicle or treatment agent, tubes were taken off nutator and worms were allowed to settle to the bottom of each tube for approximately 3 min and the supernatant was removed to a point where worms were diluted to a ratio of approximately one part worms to two parts vehicle or treatment agent solution. Then, 4 ul aliquots, containing approximately 40–80 worms, were pipetted into the center of each well of a 6-well testing plate and excess liquid was removed from the worms using a Kimwipe. Images of each well were taken 10 and/or 30 min after placing worms on test plates. It should be noted that although 0.97 mM HCl controlled for the HCl ions in the 10 mM naltrexone, there was an osmotic difference between the vehicle control and naltrexone exposure group and washing with water may induce osmotic stress. Thus, control experiments were conducted examining locomotor behavior (body bends) and movement to control attractants (benzaldehyde and food) to determine if such treatments affect either locomotor activity or normal chemotaxis to other attractants. Moreover, we (and others) have conducted washings with diH2O without effects on locomotion or the ability to develop and display learned responses to cues or preference responses to benzaldehyde (Law et al., 2004; Musselman et al., 2012; Katner et al., 2016). As with our previous work (Musselman et al., 2012; Katner et al., 2016) we are interested in only counting worms in the target zones because it provides comparable measures of elective responses of animals moving into zones that contain either the SOA or the vehicle. The vehicle zone controls for the application of a substance of the same volume as the SOA target zone and effects of that application to that space on the agar.

### SOA Preference Testing Procedure

In general, 4 μl of vehicle and a SOA solution were applied to the center of the 1.2 cm target zones of each well. These spotting solutions were allowed to absorb into agar for 30 min prior to testing. Cocaine preference was tested with 0, 50, 250, and 500 μM cocaine HCl concentrations. Nicotine preference was tested with 0, 5, 50, and 100 mM nicotine concentrations. Vehicle (water) and inhibitor agent solutions were prepared fresh, prior to each day of testing. All concentrations of SOAs include the salt and each experiment was conducted over 2 to 4 days.

### Food and Benzaldehyde Preference

Food: 1 μl of water or food (NA22 bacterial solution) was spotted to the two target zones of each well. Images were taken at 30 min. Benzaldehyde: 2 μl of a 1%(v/v) benzaldehyde solution dissolved in 25%(v/v) EtOH was spotted in one target zone, while 25% EtOH was spotted in the opposite target zone, 30 min before testing. Images were taken at 30 min. The amounts/concentrations of food and benzaldehyde were selected to produce preference indices similar to those observed with cocaine and nicotine in this paradigm.

### Nonanone Aversion

**FIGURE 1:**
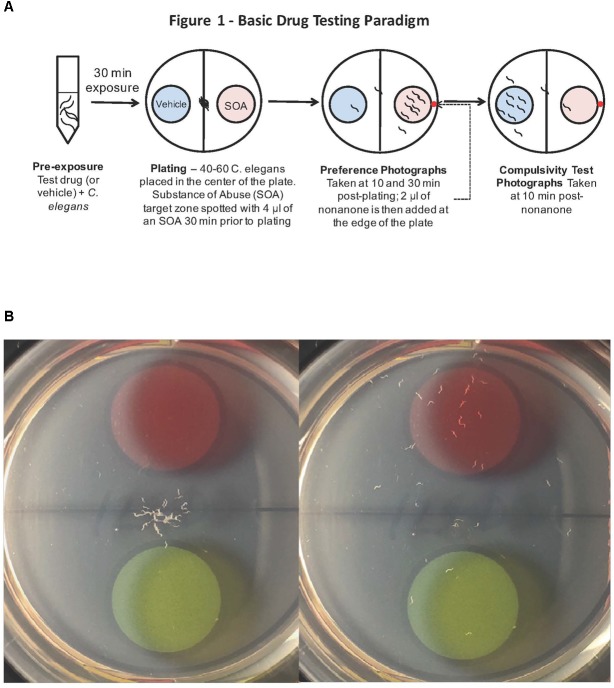
**(A)** *Caenorhabditis elegans* screen for medications development. A popular method to assess how *C. elegans* respond behaviorally to a chemical is the simple chemotaxis assay, which is a type of voluntary self-exposure paradigm. Worms were exposed to either vehicle (0.97 mM HCl) or test treatment agent (naltrexone or varenicline) in a centrifuge tube for 30 min prior to the SOA preference testing to either cocaine or nicotine. Animals were then pipetted into the center of each well of a 6-well agar test plate containing two target zones; one spotted with SOA and the other with vehicle. Images were taken 30 min after plating worms to determine a SOA preference index for each well. The chemotaxic (preference/avoidance) index (CI) is calculated by dividing the number of worms in the target zone containing the test substance by the total sum of worms counted in both zones. To indicate that the animals are not simply anesthetized by the SOA, after the preference test has been established, a 2 μl drop of the aversive stimulus nonanone (red dot) is placed between the edge of the plate and the target zone and another photograph is taken 5 min later to assess ability to determine if the presence in the target zone is the result of anesthesia. **(B)** A single well of a 6-well plate spotted with 1% benzaldehyde (red target zone) and vehicle (green target zone): Images were taken immediately (left) and 30 min after (right) placing worms in the center of the well. The benzaldehyde preference index for the well on the right at the 30 min time point was 83.3%.

Nonanone (an aversive compound) was spotted [2 μl of 10%(v/v)] to the outer edge of the SOA target zone of each well (i.e., between the edge of the SOA target zone and the outer edge of the well; see **Figure 1**) immediately after taking 30 min images for SOA preference experiments in order to determine if animals were capable of moving away from the SOA target zones and were not rendered ataxic by the SOA test compounds themselves. Therefore, images were taken immediately before and 10 min after placing nonanone into each well. Pre- and post-nonanone preference indexes (PIs) (as described below under SOA preference testing) were calculated for each well in order to compute the change in preference from the SOA target zone in response to nonanone. In this way the effects of nonanone are tested at the time and under the conditions in which the animals are displaying the preference response.

### Body Bend Assay

The body bend assay used here was adapted from Hart (2006). After 30 min pretreatment with vehicle (0.97 mM HCl) or naltrexone HCl (10 mM), 2 μl of worms diluted in a ratio of one part worms to two parts vehicle or inhibitor agent (as described above in the pretreatment section) were placed on a microscope slide on the stage of a microscope (Bausch & Lomb ASZ45L3 45X). After selecting a single worm to track, the number of times the worm’s tip crossed this midline and extended to about a 45–90 degree arc over a 20 s period was recorded. Only instances where the midline was completely crossed were counted.

### Imaging and Worm Counting

Worms were imaged by taking pictures or video with a smartphone positioned on top of a light box, which emitted light indirectly and underneath each 6-well plate. Video time-course files were compressed to time-lapse.mov files to illustrate worm activity during preference tests (± pretreatment with naltrexone) and the response to nonanone. Individual images were analyzed using ImageJ software to count the number of worms in the target zones of the test plates. Using ImageJ, the target zone was cropped from each photo and the color threshold of the image was adjusted. Specifically, threshold color was set to red, color space was set to RGB, and color threshold was adjusted so worms were highlighted in red. Particles were analyzed with a pixel size of 80 to infinity. The number of worms counted in each target zone was recorded and analyzed in Microsoft Excel.

A chemotaxic PI for each SOA concentration was then calculated by dividing the number of worms in the SOA target zone by the total sum of worms counted in both the SOA and vehicle zones converted to a percentage.

### Statistical Analyses

All analyses were analyzed using one-, two-, or three-way ANOVAs, followed by decomposition of factors and *post hoc* tests as appropriate and previously conducted (Musselman et al., 2012; Katner et al., 2016). Independent or paired *t*-tests were used to compare two samples between or within groups, respectively. Values in figures and tables are presented as mean ± SEM.

## Results

### Cocaine/Naltrexone

**FIGURE 2:**
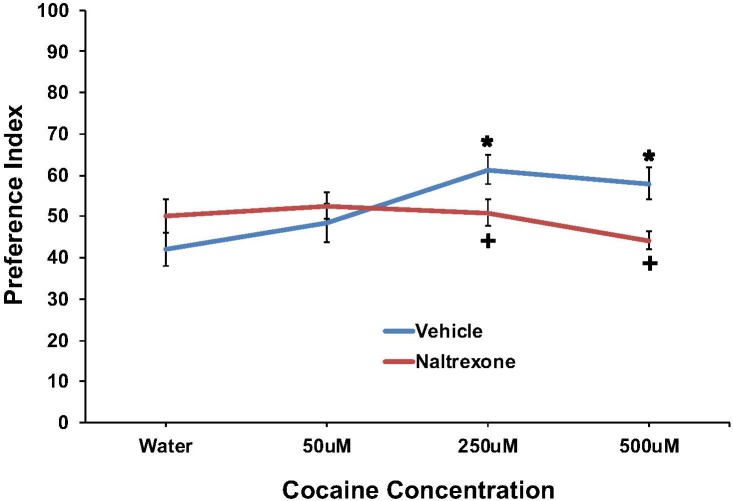
Pretreatment with 10 mM naltrexone decreased cocaine preference in N2 *C. elegans*. A two-way ANOVA found a main effect of cocaine concentration and a significant interaction between pretreatment and cocaine concentration on cocaine preference. Significant factors were decomposed using one-way ANOVAs followed by LSD *post hoc* tests. **^∗^**Significant (*p* < 0.05) increase in cocaine preference in vehicle treated worms compared to respective 0 mM cocaine (water). **^+^**Significant (*p* < 0.05) decrease in preference for cocaine following 10 mM naltrexone pretreatment compared to respective vehicle condition. The number of wells analyzed for the vehicle treated groups were 24 (water), 18 (50 μM), 18 (250 μM), and 18 (500 μM), and for the naltrexone treated groups were 24 (water), 24 (50 μM), 24 (250 μM), and 24 (500 μM).

The effect of cocaine (0, 50, 250, or 500 μM) to induce preference and the effect of pretreatment with 10 mM naltrexone to inhibit the response are shown in **Figure 2**. Preference was found for cocaine at the 250 and 500 μM concentrations, which was eliminated by naltrexone pretreatment. A two-way ANOVA found a main effect of cocaine concentration [*F*(3,174) = 2.7; *p* < 0.05] and a significant interaction between pretreatment and cocaine concentration [*F*(3,174) = 4.4, *p* < 0.006] on cocaine preference. For vehicle pretreated worms, a one-way ANOVA found a main effect of cocaine concentration on preference [*F*(3,77) = 5.0; *p* < 0.004], and *post hoc* tests revealed significant (*p* < 0.05) preference indices for 250 and 500 μM cocaine compared to water. *Post hoc* tests also found significant (*p* < 0.05) differences in cocaine preference between the 50 μM and 250 μM cocaine concentrations, for vehicle treated worms. For naltrexone pretreated worms, a one-way ANOVA did not find a main effect of cocaine concentration on preference [*F*(3,95) = 1.3; ns]. One-way ANOVAs examining differences between vehicle and naltrexone pretreatment for each cocaine concentration found no main effect of pretreatment on water [*F*(1,47) = 2.0; ns] or 50 μM cocaine [*F*(1,41) = 0.6; ns] preference. However, naltrexone pretreatment decreased 250 μM [*F*(1,41) = 4.8, *p* < 0.04] and 500 μM [*F*(1,41) = 10.8, *p* < 0.003] cocaine preference compared to vehicle pretreatment.

### Nicotine/Naltrexone

**FIGURE 3:**
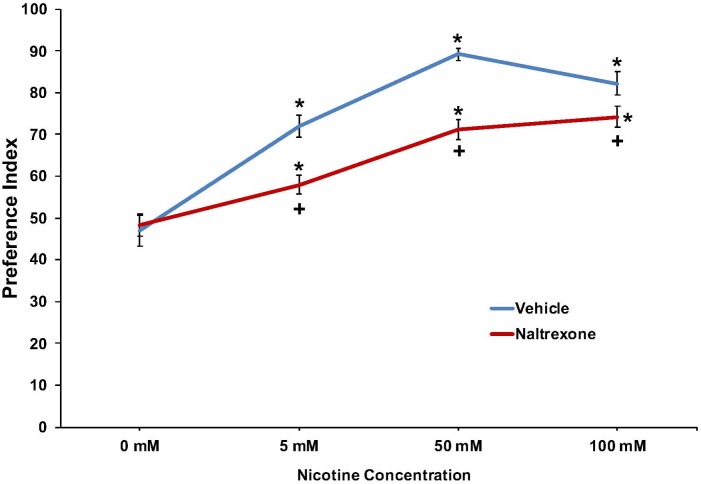
Pretreatment with 10 mM naltrexone decreased nicotine preference in N2 (wild-type) *C. elegans*. For naltrexone, a two-way ANOVA decomposed and followed by LSD *post hoc* tests where appropriate found main effects of concentration and treatment, and a significant interaction between concentration and treatment on nicotine preference at 30 min. **^∗^**Significant (*p* < 0.05) increase in nicotine preference in vehicle and naltrexone treated worms compared to respective 0 mM nicotine. **^+^**Significant (*p* < 0.05) decrease in preference for nicotine following 10 mM naltrexone pretreatment compared to respective vehicle condition. The number of wells analyzed for the vehicle treated groups were 36 (0 mM), 36 (5 mM), 36 (50 mM), and 24 (100 mM), and for the naltrexone treated groups were 36 (0 mM), 36 (5 mM), 36 (50 mM), and 24 (100 mM).

The effect of nicotine (0, 5, 50, or 100 mM) to induce a preference response and the effect of pretreatment with 10 mM naltrexone to inhibit the response is shown in **Figure 3** and **Supplementary Figure 1**. For naltrexone, a two-way ANOVA found main effects of concentration [*F*(3, 263) = 65.0; *p* < 0.001] and treatment [*F*(1,263) = 25.3; *p* < 0.001], and a significant interaction between concentration and treatment [*F*(3,263) = 5.4; *p* < 0.002] on nicotine preference at 30 min. Overall, the findings indicate a significant preference response at each nicotine concentration that is significantly reduced by pretreatment with naltrexone (**Figure 3** and **Supplementary Figure 1**).

### Nicotine/1 mM Varenicline

**FIGURE 4:**
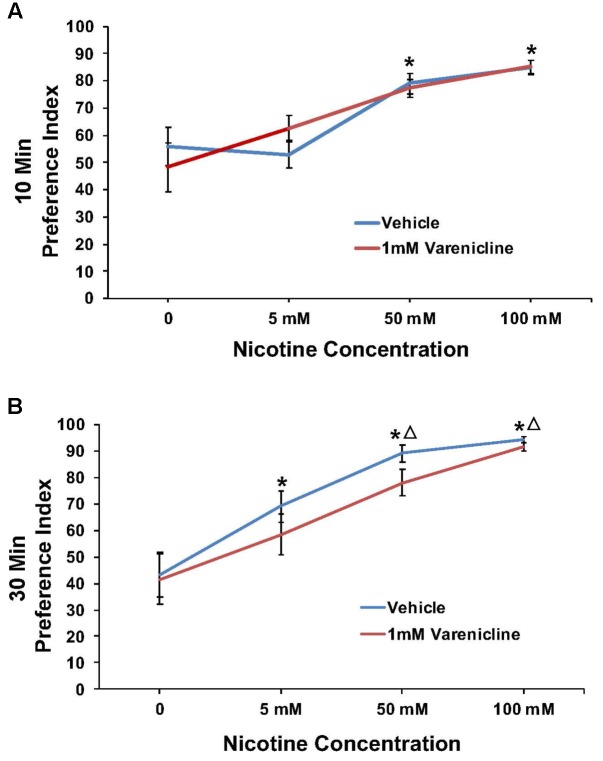
Pretreatment with 1 mM varenicline failed to modify nicotine preference in N2 (wild-type) *C. elegans*. **(A,B)** Represent 10 and 30 min nicotine preference, respectively. A two-way ANOVA decomposed and followed by LSD *post hoc* where appropriate, found a main effect of nicotine concentration on nicotine preference at 10 and 30 min. There was however, no effect of 1 mM varenicline pretreatment on nicotine preference at either time-point or nicotine concentration (*p* > 0.05). **^∗^**Significant (*p* < 0.05) increase in nicotine preference in vehicle and varenicline treated worms compared to respective 0 mM nicotine. ^Δ^Significant (*p* < 0.05) increase in preference for nicotine compared to respective 5 mM nicotine condition. The number of wells analyzed for the vehicle treated groups were 12 (0 mM), 12 (5 mM), 12 (50 mM), and 9 (100 mM), and for the varenicline treated groups were 12 (0 mM), 12 (5 mM), 12 (50 mM), and 11 (100 mM).

Varenicline pretreatment revealed robust effects at the 10 min time point in some cases, thus preference data were analyzed and presented for both the 10 and 30 min time points. The effect of pretreatment with 1 mM varenicline to inhibit the nicotine preference response is shown in **Figure 4** at both the 10 min (A) and 30 min (B) time points. For 1 mM varenicline, a two-way ANOVA found a main effect of nicotine concentration [*F*(3,91) = 23.9; *p* < 0.001] on nicotine preference at 10 and 30 min. There was however no effect of 1 mM varenicline pretreatment on nicotine preference at either time-point or nicotine concentration (*p* > 0.05).

### Nicotine/9 mM Varenicline

**FIGURE 5:**
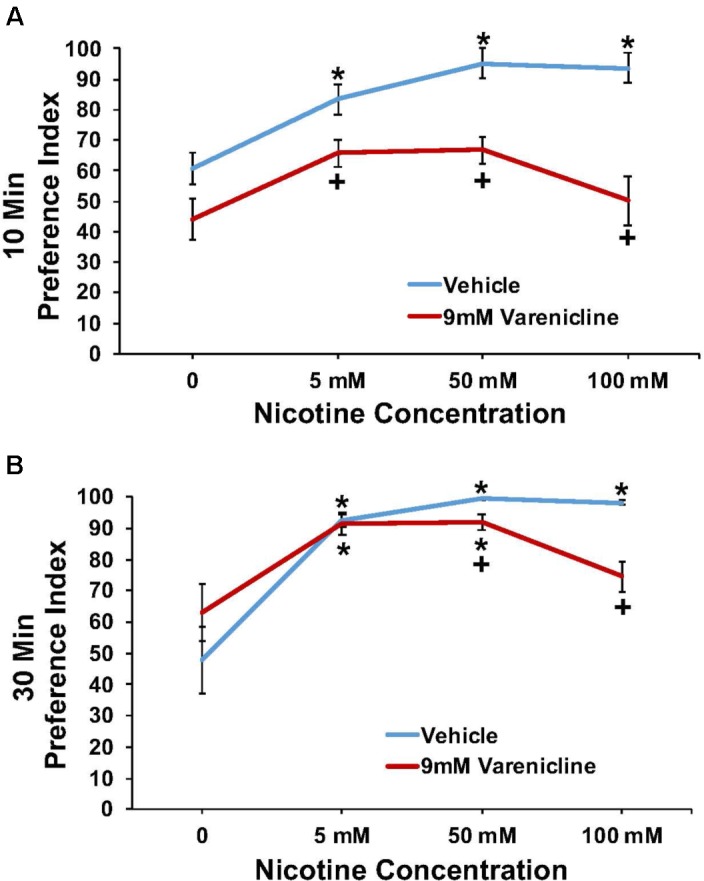
Pretreatment with 9 mM varenicline decreased nicotine preference in N2 (wild-type) *C. elegans*. **(A,B)** represent 10 and 30 min nicotine preference, respectively. A three-way ANOVA decomposed and followed by LSD *post hoc* tests where appropriate, found main effects of time (10 and 30 min), concentration, treatment, a significant interaction between concentration and treatment, and a significant interaction between time and treatment on nicotine preference. **^∗^**Significant (*p* < 0.05) increase in nicotine preference in vehicle and varenicline treated worms compared to respective 0 mM nicotine. **^+^**Significant (*p* < 0.05) decrease in preference for nicotine following varenicline pretreatment compared to respective vehicle condition. The number of wells analyzed for the vehicle treated groups were 12 (0 mM), 12 (5 mM), 10 (50 mM), and 11 (100 mM), and for the varenicline treated groups were 11 (0 mM), 12 (5 mM), 12 (50 mM), and 12 (100 mM).

The effect of pretreatment with 9 mM varenicline to inhibit the nicotine preference response is shown in **Figure 5** at both the 10 min (A) and 30 min (B) time points. A three-way ANOVA found main effects of time (10 and 30 min) [*F*(1,84) = 20.4; *p* < 0.001], concentration [*F*(3,84) = 24.5; *p* < 0.001], treatment [*F*(1,84) = 65.0; *p* < 0.001], a significant interaction between concentration and treatment [*F*(3,84) = 5.2; *p* < 0.003], and a significant interaction between time and treatment [*F*(1,84) = 16.3; *p* < 0.001] on nicotine preference. Overall, pretreatment with 9.0 mM varenicline significantly reduced the preference response to nicotine at both time points.

### Benzaldehyde Preference

The effect of pretreatment with 10 mM naltrexone to modify benzaldehyde preference was conducted in order to examine the effect naltrexone on the preference response to a known volatile attractant. An independent *t*-test found that naltrexone (10 mM) pretreatment had no significant effect [*t* = 0.97; ns] on 1% (v/v) benzaldehyde preference (PI = 84.4 ± 4.0%; *n* = 12) compared to vehicle (0.97 mM HCl) pretreatment (PI = 84.9 ± 8.6%; *n* = 12).

### Nonanone Aversion

**FIGURE 6:**
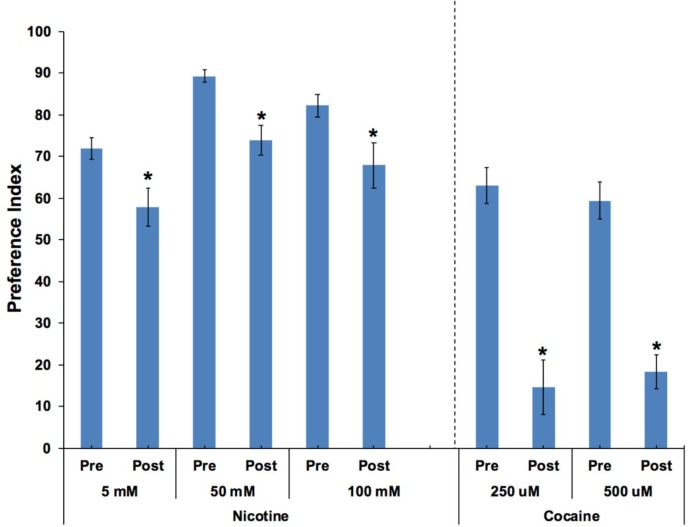
*Caenorhabditis elegans* retained the ability to move away from the aversive compound, nonanone. Nonanone application significantly reduced the preference value for nicotine (5, 50, and 100 mM) or cocaine (250 and 500 μM). Independent *t*-tests found significant effects of nonanone on 250 and 500 μM cocaine preference (*p* < 0.001). Paired *t*-tests found significant effects of nonanone on 5, 50 and 100 mM nicotine preference (*p* < 0.05). **^∗^**Significant (*p* < 0.05) decrease in SOA preference post nonanone compared to respective pre-nonanone preference for a given SOA concentration. For nicotine, the number of wells analyzed for the pre-nonanone condition were 36 (5 mM), 36 (50 mM), and 24 (100 mM), and for the post-nonanone condition were 36 (5 mM), 36 (50 mM), and 24 (100 mM). For cocaine, the number of wells analyzed for the pre-nonanone condition were 12 (250 μM) and 12 (500 μM), and for the post-nonanone condition were 6 (250 μM) and 6 (500 μM).

In order to determine if animals were anesthetized after moving into target zones containing either cocaine or nicotine, the aversive compound nonanone was applied between the target zone and the edge of the plate after the animals had established a preference response. Independent *t*-tests found significant effects of nonanone on 250 and 500 μM cocaine preference (*p* < 0.001; **Figure 6**). Paired *t*-tests found significant effects of nonanone on 5, 50, and 100 mM nicotine preference (*p* < 0.05; **Figure 6**).

### Video of SOA Preference, the Response to Nonanone, and the Effect of Naltrexone Pretreatment

To better illustrate the development of drug preference and the aversive response to nonanone in this paradigm, videos of a preference test with 100 mM nicotine and the subsequent response to nonanone were recorded and compressed into time-lapsed format and are presented in **Supplementary Video 1**. A similar time-lapsed video illustrating the effects of pretreatment with 10 mM naltrexone on the preference test with 100 mM nicotine is presented in **Supplementary Video 2**.

### Food Preference

For vehicle and naltrexone pretreatment, both conditions showed a normal chemotaxis to food and food preference was 86.1 ± 2.8 and 80.3 ± 5.7%, respectively (**Table 1**). An independent *t*-test found no significant effect of pretreatment (vehicle vs. naltrexone) on food preference [*t* = 2.1; ns]. In addition, 9 mM varenicline pretreatment did not significantly affect food preference (**Table 1**). An independent *t*-test found no effect of varenicline on food preference [*t* = 2.2; ns].

**Table 1:** Food preference in N2 *C. elegans* following vehicle and inhibitor agent treatment.

Agent	Vehicle	Inhibitor Treated
Naltrexone (10 mM)	86.1 ± 2.8%	80.3 ± 5.7%
Varenicline (9 mM)	84.0 ± 3.6%	80.7 ± 5.0%

*t-tests found that inhibitor agent treatments had no effect on food preference. The number of wells analyzed for naltrexone were 12 for vehicle and 12 for naltrexone. The number of wells analyzed for varenicline were 12 for vehicle and 12 for varenicline.*

### Locomotor Activity

Naltrexone: We found that exposure to 10 mM naltrexone for 30 min prior to testing had no significant effect on locomotor activity compared to vehicle exposure [*F*(1,11) = 0.02; ns]. Specifically, the number of body bends in 20 s for vehicle and naltrexone treatments were 54 ± 7 (*n* = 6) and 53 ± 3 (*n* = 6), respectively.

Varenicline: Mean (±SEM) body bends/20 s for vehicle treated worms were 46.3 ± 3.6 (*n* = 9), while body bends were 47.0 ± 3.6 (*n* = 8) for varenicline (9 mM) treated worms. Independent *t*-tests found no significant difference in body bends after varenicline treatment.

## Discussion

The present studies found a concentration-dependent attraction by *C. elegans* to the SOAs cocaine (**Figure 2**) and nicotine (**Figures 3–5** and **Supplementary Video 1**). Naltrexone pretreatment selectively reduced preference for both cocaine (**Figure 2**) and nicotine (**Figure 3**, **Supplementary Figure 1** and **Supplementary Video 2**) in this paradigm, but had no effect on preference for food or benzaldehyde preference. Moreover, the SOA preference response was not likely due to an anesthetic or paralytic effect, since worms were able to move away from the SOA target zones following the application the chemorepellent, nonanone (**Figure 6** and **Supplementary Video 1**), and continue to move in the SOA target zone after entering (**Supplementary Video 1**). Pretreatment with varenicline, a treatment agent approved for smoking cessation in humans, also was found to reduce the nicotine preference response at concentrations that did not affect locomotor activity or food preference. These data are consistent with observations in vertebrate animal models showing efficacy and selectivity of the SOAs and begins to provide face and predictive validity for the model in medications screening applications. Importantly, the 6-well plates enable a high-throughput system for behavioral screening, and are able to reduce the number of *C. elegans* needed to conduct preference testing. This also limits the time required for imaging, and ultimately enhances throughput. Combined, these findings suggest that procedures using *C. elegans* may be developed to screen medications for the treatment of substance use disorders. In addition, the development of this technology will allow for the future investigation of the molecular mechanisms that underlie the efficacious effects of novel agents using the fully tractable *C. elegans* model.

A popular method to assess how *C. elegans* respond behaviorally to a chemical or substance is the simple chemotaxis assay (Bargmann and Horvitz, 1991; Bargmann, 2006) which, in fact, is a type of voluntary self-exposure paradigm. In the current studies, we employed a modified version of this assay, in which a 6-well agar test plate was prepared with a SOA placed in a defined target region on one side of each well and the vehicle, usually water, placed in a target zone on the other side of each well. The current experiments build on our previous work showing that *C. elegans* show conditioned attraction to cues (either a salt or food cue) previously paired with cocaine or methamphetamine which utilized a procedure analogous to Pavlovian conditioning models of reward in rodents (Musselman et al., 2012; Katner et al., 2016). The current work examines preference responses to two of the most widely abused stimulants, cocaine and nicotine (Lee et al., 2009; Ward et al., 2009; Musselman et al., 2012; Sellings et al., 2013). Few studies have examined the reinforcing properties of stimulants in *C. elegans*; however, Sellings et al. (2013), demonstrated that *C. elegans* show a concentration dependent attraction to nicotine applied to agar test plates which were confirmed in the current findings with nicotine. It should be noted that the concentrations of treatment agents needed to produce effects in *C. elegans* in these studies and in the current study are often high due to the waxy cuticle that encases the animal and functions as a barrier to entry (Epstein, 1995; Davies et al., 2003; Cheong et al., 2015). In the current work, animals counted in the target zone containing the stimulant (either cocaine or nicotine) are in contact with the SOA and thus demonstrating self-exposure to the SOA. This is also a true choice behavior, since the current study found that the addition of the aversive compound nonanone near the SOA target zone, after the preference response has been established, caused the animals to immediately move away from the SOA target zone, inducing a measurable aversive response. These findings confirm that the SOAs tested here are not simply functioning as a simple locomotor anesthetic or paralytic agent in this procedure.

Consistencies in responses to SOAs across phyla led to the hypothesis that *C. elegans* may be a viable model system to screen potential candidate treatment for substance use disorders. Recently, *C. elegans* were found to have functional opioid-like receptors (Cheong et al., 2015). Thus, to determine the predictive validity of the model, we tested the effectiveness of naltrexone to decrease preference responses, as it is one of the very few compounds shown consistently to reduce alcohol and other SOAs intake and seeking behavior in animal models as well as humans (Heilig and Egli, 2006). Using vertebrate models, naltrexone has been demonstrated to reduce cocaine intake (Mello et al., 1990; Corrigall and Coen, 1991; Ramsey and van Ree, 1991) and seeking (Giuliano et al., 2013); opioid intake in animal models and humans (Negus and Banks, 2013), and has recently been shown to decrease cannabis self-administration and subjective effects in chronic cannabis users (Haney et al., 2015). Naltrexone has shown mixed effects on nicotine use in humans (Aboujaoude and Salame, 2016; Barboza et al., 2016; Kirshenbaum et al., 2016). Rodent studies indicate that naltrexone can reduce nicotine-induced locomotor sensitization (Goutier et al., 2016) self-administration at 2.0 mg/kg (Guy et al., 2014) but not 1.0 mg/kg or below (Le et al., 2014). Also, treatment with naloxanazine (a selective mu1 opioid receptor antagonist) significantly reduced nicotine self-administration in rats (Liu and Jernigan, 2011). However, some work suggests that naltrexone may have more consistent effects to reduce conditioned responses to nicotine (Liu et al., 2009). Other opioid receptors may also be efficacious targets, with the kappa-opioid receptor antagonist nor-binaltorphimine reducing nicotine seeking behavior (Grella et al., 2014). Together, these studies support a role for opioid systems in stimulant reinforcement and use and are consistent with findings in the current screen with *C. elegans*. However, much additional investigation is needed to identify how the opioid system may be involved in nicotine self-administration and how agents that target these systems may reduce tobacco or cocaine use in humans.

Based on the data presented here, we anticipate that potential compounds that have efficacy in reducing SOA intake and/or seeking in vertebrate models and humans will also inhibit the SOA preference response in *C. elegans*. Naltrexone pre-exposure clearly reduced SOA preference (**Figures 2**–**3**) at concentrations that do not inhibit food consumption (**Table 1**), benzaldehyde chemotaxis, or locomotor activity (body-bend data). These data are consistent with rodent data showing that naltrexone can inhibit intake of SOAs at doses that do not affect sucrose intake or body weight (Henderson-Redmond and Czachowski, 2014). In most instances, little or no prior work has been published to determine if treatment agents used to treat stimulant addictions have effects on models of addictive responses to stimulants in *C. elegans*. However, varenicline pre-exposure has been shown to reduce chemotaxis to nicotine in *C. elegans* (Sellings et al., 2013). Our data are consistent with these data and show selectivity and predictive validity of varenicline in this screening model. Varenicline is a partial agonist at the α4β2 receptor in vertebrates and is an approved treatment for nicotine addiction (Crooks et al., 2014). Although it is still unclear how varenicline reduces nicotine preference in *C. elegans*, attraction to nicotine may be mediated through the acr-5 and acr-15 nicotinic acetylcholine receptors (Sellings et al., 2013). Other possible mechanisms such as changes in drug metabolism or subtle changes in sensory systems have yet to be investigated. Interestingly, the effect of varenicline to inhibit nicotine self-exposure in this paradigm is evident at the 10-min time point and, although still evident at 30 min, appears to degrade over time (**Figrue 5**). This could be a reflection of the apparent strength of the nicotine preference response, or possibly rapid clearance of varenicline. In support of this idea is the apparent greater strength of the preference response, and resistance to nonanone for nicotine compared with cocaine at the concentrations used in this study (**Figure 6**). Although it is somewhat difficult to make direct comparisons between the cocaine and nicotine data due to the differences in systems and mechanisms, and also in concentrations used to produce the respective preference responses, there are clear differences in the response to nonanone. One possible explanation is that animals are being paralyzed by the SOAs at these concentrations. This cannot be completely ruled out as previous work has indicated that nicotine uniformly mixed in agar to concentrations from 1 to 10 mM can induce paralysis (Sobkowiak et al., 2011). However, other evidence argues against the idea that the worms are paralyzed. First, although the concentrations of cocaine and nicotine contacting the worms in the target zones are not known, only 4 μl of SOA was absorbed into a target zone in a well containing 3.8 mls of agar, indicating the concentrations contacting the worms was likely much lower than the concentrations added to the target zones. Secondly, both groups of animals show a significant effect of nonanone to move the animals from the SOA target zone (although the magnitude of the effect was less for nicotine), indicating that they are not paralyzed. This hypothesis is further supported by examination of time-lapse videos (**Supplementary Video 1**) which clearly show animals continuing to move in the nicotine target zone after entering during a preference test on a plate spotted with 100 mM nicotine, and a clear movement out of the zone after the addition of nonanone. One possible explanation for the greater effect of nonanone to displace cocaine exposing animals compared to nicotine is the somewhat stronger preference response observed with nicotine vs. cocaine in these assays (**Figure 6**). The increased preference response for nicotine over cocaine suggests a greater reinforcing property of the SOA as tested and as such would confer greater aversion resistance. Future experiments will provide additional evaluation and characterization of varenicline and other compounds to inhibit the SOA preference response. Such work is needed to further demonstrate predictive validity and provide a strong case for the model’s utility as a screening tool to help identify compounds that have potential as treatments for SOA and alcohol use disorders.

Behavioral studies of addictive SOAs in *C. elegans* to date have mostly focused on EtOH (Grotewiel and Bettinger, 2015; Engleman et al., 2016). Additional studies with other SOAs are needed to better characterize the mechanisms that underlie addictive properties of SOAs across the many classes of SOAs and how they may be consistent or divergent across species. In the few studies conducted thus far, several molecular targets have been identified in various behavioral paradigms across SOA classes using *C. elegans* (Engleman et al., 2016). Thus far, it appears that genes involved in monoamine neurotransmission mediate at least some behaviors induced by each SOA (Bettinger and McIntire, 2004; Lee et al., 2009; Ward et al., 2009; Musselman et al., 2012; Sellings et al., 2013; Topper et al., 2014; Matsuura and Urushihata, 2015). In particular, mutation of the gene coding for tyrosine hydroxylase (*cat-2*) reduced or inhibited SOA-induced behaviors for each SOA of abuse (Bettinger and McIntire, 2004; Musselman et al., 2012; Matsuura and Urushihata, 2015). It is widely thought that the dopamine neurotransmitter system plays an important role in drug abuse (Koob, 1992; Koob and Volkow, 2010) and all of the SOAs discussed here have effects on dopamine neurotransmission. Similarly, in agreement with the current data, previous work has shown that manipulations that inhibit cholinergic neurotransmission in *C. elegans* will also affect the behavioral response to nicotine (Feng et al., 2006; Sellings et al., 2013) and the effects of varenicline may be due in part to its effect in modulating dopamine neurotransmission (Crooks et al., 2014). Overall, the known mechanisms of action of SOAs in vertebrate animals thus far show parallel findings in *C. elegans* and other invertebrates. The effects of SOAs in *C. elegans* appear likely to be mediated by neurobiological systems associated with many genes and proteins that are known to mediate and/or support neuronal function in higher level organisms (for review see Engleman et al., 2016). However, since most of the work in *C. elegans* thus far has focused heavily on EtOH, additional studies are needed to determine if these mechanisms are also involved in other SOAs using the *C. elegans* as a model system. Since the contributions of the olfactory/chemosensory systems and specific mechanisms of attraction and self-exposure will likely changes across various classes of drugs of abuse, such differences may provide further insight into the addictive properties of individual drugs and will be a key focus of future studies.

Although *C. elegans* phenotypes are surprisingly highly conserved functionally, with few clear differences in neurobiology, pharmacology, and molecular systems between vertebrates and *C. elegans*. Moreover, *C. elegans* simple nervous system lacks the complex neurocircuitry of mammals that have been found to be involved in addiction (Koob and Volkow, 2010). However, the similarities in responses to SOAs between *C. elegans* and mammals suggests that the behavioral responses to SOAs may rely more on functional similarities in terms of how SOAs affect systems that mediate survival (food) of the species rather than complexities in the neuroanatomy. Interestingly, differences in receptor systems and molecular pharmacology in *C. elegans* could also provide an advantage of this model providing a unique perspective concerning how some classes of putative treatments affect SOAs. As an example, topiramate is under investigation as a possible treatment for EtOH use disorders (Johnson, 2004). Topiramate has a rich pharmacology and there are several possible molecular mechanisms for reducing EtOH drinking behavior. One suggested mechanism is activity at voltage-sensitive sodium channels (Johnson, 2004), which are not present in *C. elegans* (Bargmann, 1998). If topiramate were to be found ineffective in *C. elegans* assays of EtOH self-exposure, the data would support the contention that sodium channels may have a role in reducing SOA intake/seeking in vertebrates. Thus, cross-species findings could be assessed with respect to molecular homology of the mechanisms thought to be mediating SOA taking and/or preference. This could be conducted across SOA classes to identify the effects of divergent molecular structure or function on the results. Overall, such investigations may help to characterize the molecular and pharmacological foundations of the effects of these compounds, whether or not the findings are consistent with the anticipated results.

Further development of the model employed here is anticipated to provide the field with a new and powerful tool to discover novel targets and treatments for addiction. This work will combine the advances in our knowledge of human addiction and insight gained through the use of vertebrate behavioral models, and apply them to invertebrate models with tremendous advantages and potential for discovery on a number of levels: (a) the current work contributes to the establishment of a new behavioral model in *C. elegans* for screening candidate compounds to treat stimulant addictions; (b) in addition, the ability to manipulate genes and gene expression quickly, and the availability of many mutant strains in this well studied and simple organism, greatly enhances the capability to discover specific genes and proteins involved in SOA preference behavior in this model; (c) the low cost and potential to fully automate the assays allows for a dramatic increase in the number of experiments that can be conducted for a fraction of the cost and time needed with other animal models. Thus, this model might be used in conjunction with gene editing techniques like CRISPR where *C. elegans* receptors can be replaced with their human orthologs to create transgenic *C. elegans* that might show human-like pharmacology. Such an application could improve the translational utility of the model and possibly enhance predictive validity.

Once a high-throughput system is fully established, one could conceivably screen entire potential treatment agent libraries using tiny amounts of expensive compounds relative to other animal models. Future collaborative projects will employ transgenic approaches to express human genes in this model to enhance the predictive validity of the model. Finally, the data will be bi-directionally informative with other animal models of medications development for substance use disorders and compounds in clinical trials, such that the diversity in pharmacology and molecular systems between different species will help to better identify the mechanisms of action of putative and/or validated treatment compounds.

## Author Contributions

EE and SK wrote the initial drafts and designed and directed the experiments. KS, KB, MB, and HK performed the experiments and provided critical feedback for method development and manuscript preparation. RB and BN-B reviewed content, data, and final draft of the manuscript.

## Conflict of Interest Statement

The authors declare that the research was conducted in the absence of any commercial or financial relationships that could be construed as a potential conflict of interest.
