# Supplementary material for: Caenorhabditis elegans Show Preference for Stimulants and Potential as a Model Organism for Medications Screening
Source: Front Physiol. 2018 Aug 30;9:1200. doi: 10.3389/fphys.2018.01200 (PMC6125605; doi:10.3389/fphys.2018.01200)
Supplement: Supplementary file 1 [file Table_1.DOCX]

***Supplementary Time-Lapse Video Figure Legends:***

**Link to videos:**

<https://iu.box.com/s/o7drhqtwp370lk21112khksc4mjdbqbj>

**Supplementary Video 1**. Time-lapse video of N2 adult *C. elegans* being tested for nicotine preference in a single well of a 6-well plate: In the well of a 6-well agar test plate, the red target zone was spotted with 100 mM nicotine and the green target zone with vehicle (water) 1 hour prior to preference testing. Worms were treated with pre-treatment vehicle for 30 min as described in the Methods section. Subsequently, N2 adult *C. elegans* were pipetted into the center of this well containing the nicotine and vehicle spotted zones. Time-lapse video was initiated immediately and continued for 30 min. Time-lapse video was also taken after the aversive compound nonanone (10%) was applied near the nicotine target (red) zone beginning at the 31 min time point. The development of nicotine preference can be observed up to the 30 min time point. After the presentation of the aversive compound nonanone at the 31 min time point, worms clearly move away from the nicotine target zone, illustrating (1) the development of the nicotine preference response, (2) continued movement within the nicotine target zone showing that the preference is not due to a simple anesthetic effect of nicotine, and (3) movement away from nicotine target zone in response to application of the aversive compound nonanone. See methods section for additional details.

**Supplementary Video 2**. Time-lapse video of N2 adult *C. elegans* being tested for nicotine preference in a single well of a 6-well plate after pre-exposure to 10 mM naltrexone: In the well of a 6-well agar test plate, the red target zone was spotted with 100 mM nicotine and the green target zone with vehicle (water) 1 hour prior to preference testing. Animals were pre-exposed to 10 mM naltrexone for 30 min prior to testing as described in the Methods section. Subsequently, N2 adult *C. elegans* were pipetted into the center of this well containing the nicotine and vehicle spotted zones. Time-lapse video was initiated immediately and continued for 30 min. Time-lapse video was also taken after the aversive compound nonanone (10%) was applied near the nicotine target (red) zone beginning at the 31 min time point. The pretreatment with naltrexone reduced nicotine preference at the 30 min time point. It should be noted that naltrexone pretreatment had no significant effect on preference for either food (**Table 1**) or the attractant benzaldehyde at concentrations that produced similar preference scores. After the presentation of the aversive compound nonanone at the 31 min time point, worms show movement away from the nicotine target zone.
