# Supplementary material for: Caenorhabditis elegans Show Preference for Stimulants and Potential as a Model Organism for Medications Screening
Source: Front Physiol. 2018 Aug 30;9:1200. doi: 10.3389/fphys.2018.01200 (PMC6125605; doi:10.3389/fphys.2018.01200)
Supplement: Supplementary file 2 [file Table_2.DOCX]

**Supplementary Figure 1**. Box-and-Whisker and Scatter Plots: In order to illustrate characteristics of the SOA preference data, the preference data presented in Figure 3 depicting nicotine preference and the effect of pretreatment with 10 mM naltrexone are presented in (A) Box-and-Whisker and (B) Scatter plots. Figures were generated in GraphPad Prism. Box-and-Whisker shows minimum, 25th percentile, median, 75th percentile, and maximum values. Scatter plots show all data included in the analyses and the mean for each group.
