# Supplementary figures and images for: Caenorhabditis elegans Show Preference for Stimulants and Potential as a Model Organism for Medications Screening
Source: Front Physiol. 2018 Aug 30;9:1200. doi: 10.3389/fphys.2018.01200 (PMC6125605; doi:10.3389/fphys.2018.01200)

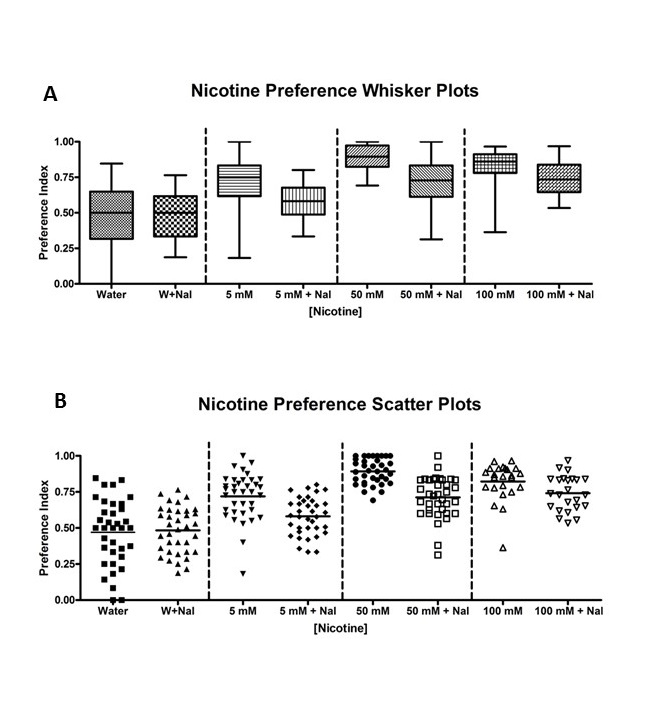

Supplement: Supplementary file 3 [file Image_1.JPEG]
